# Bakers' Exhibition

**Published:** 1906-09-15

**Authors:** 


### THE BAKERS' EXHIBITION.

The Bakers' Exhibition held during this week at the Agricultural Hall, Islington, surrounds the above article with particular interest to every part of the country. The Scottish Section is exceptionally strong, and their devotion to the confectioners' art should receive ample demonstration at this meeting. We notice, however, that for real artistic work the valuable prizes fall to Englishmen. One of the most interesting exhibits apart from the magnificent shows of the great milling and flour-producing firms is that of Bananine and flour made by a specially devised process from green bananas. It is a delightful preparation, making well up into splendid wafers. They are supplied to the King of Spain and other eminent persons.

The nutritive properties are so well known that the bread produced from it is highly nourishing, very suitable for invalids, and possesses also laxative qualities on account of the natural fruit syrups it contains. It is readily digested and highly suitable for people who have to lead sedentary lives. It is smooth, not so irritating as brown bread. It is a highly concentrated preparation, eleven pounds of pure banana fruit being required to make one pound of flour. The "Lucerna" double milk chocolate is another attractive novelty at this great exhibition. It is prepared by a special process which renders it more digestible. It is of a particularly fine quality. The whole place is full of the aroma of scented sweetmeats and the scent of bread-making, and affords a grand object-lesson on the value of deftness and cleanliness in the processes of food-making.

